# Multivariate Trajectories of Eating Disorder Symptoms and Weight Status in 10‐ to 17‐Year‐Old Children and Adolescents

**DOI:** 10.1002/eat.70045

**Published:** 2026-02-06

**Authors:** Anja Hilbert, Danielle Schewe, Andreas Hiemisch, Antje Körner, Wieland Kiess, Ricarda Schmidt

**Affiliations:** ^1^ Integrated Research and Treatment Center AdiposityDiseases, Behavioral Medicine Research Unit, Department of Psychosomatic Medicine and Psychotherapy, Leipzig University Medical Center Leipzig Germany; ^2^ German Center for Child and Adolescent Health (DZKJ), partner site Leipzig/Dresden Leipzig Germany; ^3^ LIFE Leipzig Research Center for Civilization Diseases, Leipzig University Leipzig Germany; ^4^ Department of Women and Child Health University Hospital for Children and Adolescents and Center for Pediatric Research, Leipzig University Leipzig Germany

**Keywords:** binge eating, body mass index, compensatory behavior, development, eating disorder, latent structure analysis, obesity, restraint, trajectory

## Abstract

**Objective:**

Eating disorders (EDs) often emerge in adolescence, but developmental trajectories across different core features remain largely unclear.

**Method:**

The prospective, community‐based study included *N* = 898 participants aged 9.5–17.5 years (47.6% female, age 11.8 ± 1.4 years) with annual follow‐up over 2–6 (3.4 ± 1.2) years. Multivariate trajectories of binge eating, restraint, weight‐compensatory behaviors (Eating Disorder Examination‐Questionnaire for Children), and body mass index‐standard deviation score (BMI‐SDS) derived from objective anthropometrics were analyzed using group‐based multi‐trajectory modeling (GBMTM) separately for girls and boys. ED and general psychopathology were used for validation and outcome comparisons.

**Results:**

GBMTM identified five distinct trajectories of ED symptoms and BMI‐SDS in girls and six in boys. Low‐symptom trajectories at lower, normal, and higher BMI‐SDS were most common and showed only mild, transient ED symptoms. In boys, trajectories were largely characterized by stable ED symptoms at different BMI‐SDS levels, whereas in girls, ED symptoms showed more pronounced change over time. In both sexes, two high‐risk subgroups reflected bulimic and binge‐eating patterns and followed trajectories with elevated or increasing ED symptoms across ages, which in girls were particularly associated with increased ED and general psychopathology at last assessment.

**Discussion:**

GBMTM results support the developmental specificity of bulimic/binge‐eating syndromes and noneating‐disordered overweight across adolescence. High‐risk subgroups in both sexes—and their particularly unfavorable outcomes in girls—underscore the need for sex‐specific early identification strategies that consider longitudinal constellations of multiple ED symptoms and weight status rather than single indicators.

## Key Points

- Eating disorders often begin in adolescence, but it is still unclear how symptoms like binge eating, restrictive eating, unhealthy weight control, and body weight evolve together across development.
- In 9.5–17.5‐year‐olds, this study identified five distinct trajectories in girls and six trajectories in boys, including two high‐risk patterns showing highest eating disorder psychopathology levels over time, particularly among girls.
- Monitoring changes in multiple symptoms may improve early detection and guide targeted prevention or early intervention.

## 1 Introduction

Adolescent‐onset eating disorders (EDs) affect 3%–10% of youth, including anorexia nervosa, bulimia nervosa, and binge‐eating disorder, characterized by aberrant eating behaviors and weight dysregulation (American Psychiatric Association [APA] 2013), with more girls than boys affected (Silén and Keski‐Rahkonen 2022; Swanson et al. 2011). Many more children and adolescents experience ED symptoms, beginning as early as middle childhood or early adolescence (McClelland et al. 2020). Overall, 30% of girls and 17% of boys display symptoms such as binge eating (i.e., consuming objectively or subjectively large amounts of food with a sense of loss of control over eating), compensatory behaviors to prevent weight gain (e.g., self‐induced vomiting), or restrained eating (i.e., conscious attempts to restrict food intake to control weight or shape; López‐Gil et al. 2023), emerging at different developmental windows (McClelland et al. 2020). Obesity defined as body mass index (BMI, kg/m^2^) for age ≥ 2 SD (de Onis & Onis and Lobstein 2010) affects 6.9% of girls and 9.3% of boys globally (NCD Risk Factor Collaboration [NCD‐RisC] 2024). Yet, many more children and adolescents experience excess weight gain (NCD‐RisC 2020; Schienkiewitz et al. 2019). Underweight prevalence (BMI for age < 2 SD; Onis and Lobstein 2010) amounts to 8.2% in girls and 10.8% in boys (NCD‐RisC 2024). Notably, ED symptoms and deviations in age‐adjusted BMI often co‐occur; for example, binge eating (disorder) and bulimia nervosa are more common in adolescents with obesity (López‐Gil et al. 2023; Mitchison et al. 2020). Both EDs and underweight/obesity are significant public health concerns in youth (Austin 2011; Wilksch et al. 2015) due to their profound and lasting detrimental effects on physical and mental health and social functioning (Santomauro et al. 2021).

The developmental course of ED symptoms and weight status in adolescence is complex, heterogeneous, and not yet fully understood (McClelland et al. 2020; Yamamiya et al. 2023). Limited longitudinal evidence suggested that these characteristics follow distinct but interrelated trajectories (Yoon et al. 2020). Variable‐centered longitudinal analyses (e.g., latent growth models) have shown that ED symptoms and psychopathology increase linearly up to late adolescence and then decline or stabilize into young adulthood (Davis et al. 2018; Dufour et al. 2023; Evans et al. 2019; Slane et al. 2014). While some of these studies identified similar trajectories of ED symptoms and psychopathology for boys and girls (Davis et al. 2018), others reported higher and more steeply increasing symptom levels for girls than boys from mid‐ to late adolescence (Dufour et al. 2023; Verschueren et al. 2020). Regarding age‐adjusted BMI, growth was found to accelerate during late adolescence particularly in those at higher initial body weight, while stable age‐adjusted BMI trajectories emerged for adolescents at lower and normal weight, in both girls and boys (Geserick et al. 2018). Although such variable‐centered approaches describe average trends in single symptoms, they do not capture interindividual heterogeneity in the joint developmental trajectories of ED symptoms and weight status.

To examine developmental heterogeneity, a few studies have used univariate person‐centered longitudinal latent structure analyses methods grouping adolescents into trajectory classes based on their change in single ED symptoms (Aimé et al. 2008; Bodell et al. 2018; Breton et al. 2022; Fairweather‐Schmidt and Wade 2016; Fay and Lerner 2013; Wang et al. 2019). From 10 to 15 years of age, three to four trajectories of binge eating and self‐induced vomiting or purging were identified (none, increasing, decreasing, high) in adolescent girls (Pearson and Smith 2015; Smith et al. 2007). Further, in weight‐related research, univariate person‐centered analyses have mostly identified three to four trajectory classes across childhood and adolescence, including stable average, stable high, and increasing (and decreasing) age‐adjusted BMI (Ali et al. 2022; Frank et al. 2023; Mattsson et al. 2019).

However, these univariate studies ignored covariations among ED symptoms and weight status (see Davis et al. 2018; Kansi et al. 2005). Only one study has applied a multivariate person‐centered trajectory approach to jointly examine change across multiple variables: Verschueren et al. (2020) followed 15‐year‐old adolescents for 2 years in their level of body dissatisfaction, drive for thinness, bulimic symptoms, and age‐adjusted BMI. Multivariate latent trajectory class analyses revealed four classes in girls and two in boys. Among girls, classes included a large normal‐weight/low ED symptom group, a normal‐weight/high ED symptom group, an underweight/low ED symptom group, and a smaller overweight/high ED symptom group. In boys, variability was more restricted, with one small group of boys who were slightly at higher weight and reported more ED symptoms, and a large group with normal weight and very low ED symptom levels. Notably, changes in age‐adjusted BMI accounted for more trajectory variability than changes in ED symptoms, particularly in boys.

Thus, existing studies provided initial evidence on how ED symptoms and age‐adjusted BMI develop together at the individual level. However, joint trajectories of core behavioral symptoms, such as binge eating and compensatory behaviors, and weight status across the broader span of adolescence remain insufficiently understood. Therefore, the present study aimed to examine developmental trajectories of ED symptoms and weight status across adolescence using person‐centered multivariate latent trajectory modeling, and to investigate baseline correlates and outcomes of these trajectories. Because of pronounced sex differences in ED symptoms in adolescents (Mitchison et al. 2020; Verschueren et al. 2020), all models were estimated separately for girls and boys.

## 2 Method

### 2.1 Participants

This study is part of LIFE Child‐study investigating childhood development and the etiology of lifestyle diseases in a community‐based longitudinal cohort, conducted by the Leipzig Research Center for Civilization Diseases (Poulain et al. 2017; Quante et al. 2012). The LIFE Child‐study was approved by the Ethical Committee of the Medical Faculty of the University of Leipzig (No. 264/10‐ek) and registered as a clinical trial (NCT02550236). Recruited through advertisements at public health centers, schools, and clinics in and around Leipzig, Germany, children and adolescents 24 weeks of gestation to 16 years of age were included, excluding those with chronic, chromosomal, or syndromic diseases. All parents provided written informed consent. Written assent was also obtained from the children if they were ≥ 12 years of age. Participants underwent annual follow‐up assessments to track longitudinal changes in development. A total of 4132 children and adolescents were enrolled from January 4, 2011 to December 31, 2021.

Participants were excluded if they were < 9.5 or > 17.5 years old (resulting in 2014 participants), had < 2 full assessments of the four trajectory variables (1048 participants), or had a sibling who had already participated (only the first child per family was retained), yielding 902 participants. Further, four participants were excluded for extreme values. The final sample of *N* = 898 consisted of *n* = 427 girls and *n* = 471 boys with a mean age of 11.83 ± 1.42 years and a mean body mass index standard deviation score (BMI‐SDS) of −0.09 ± 0.98. Further sociodemographic and clinical baseline characteristics of the study sample are presented in Table 1. Participants completed an average of 3.35 ± 1.18 of the yearly assessments (median: 3, range 2–6 assessments), with the following distribution: 2 (32.3%), 3 (22.5%), 4 (25.6%), 5 (16.7%), and 6 (2.9%) assessments. Mean age at last assessment was 14.45 ± 1.40 years.

**TABLE 1:** Sociodemographic and clinical sample characteristics at baseline.

	Total (*N* = 898)	Girls (*n* = 427)	Boys (*n* = 471)
	*M* (SD)/*n* (%)	*M* (SD)/*n* (%)	*M* (SD)/*n* (%)
Age, years	11.83 (1.42)	11.83 (1.45)	11.82 (1.39)
BMI‐SDS	−0.09 (0.98)	−0.06 (1.03)	−0.12 (0.93)
Weight status			
Severe underweight	27 (3.0%)	14 (3.3%)	13 (2.8%)
Underweight	60 (6.7%)	27 (6.4%)	33 (7.0%)
Normal weight	728 (81.3%)	343 (80.7%)	385 (81.9%)
Overweight	60 (6.7%)	27 (6.4%)	33 (7.0%)
Obesity	20 (2.2%)	14 (3.3%)	6 (1.3%)
Eating disorder psychopathology, ChEDE‐Q, 0–6	0.51 (0.84)	0.67 (0.97)	0.37 (0.67)
Eating disorder psychopathology, ChEDE‐Q, 0–6^a^	0.57 (0.91)	0.75 (1.05)	0.41 (0.74)
General psychopathology, SDQ self‐report, 0–40	10.04 (5.01)	10.29 (4.89)	9.82 (5.11)
General psychopathology, SDQ parent‐report, 0–40	7.30 (5.16)	7.24 (5.17)	7.36 (5.15)
Socioeconomic status			
Mean Winkler index, 3–21	13.90 (3.28)	13.82 (3.34)	13.97 (3.22)
Low SES, 3–8	56 (6.2%)	28 (6.6%)	28 (5.9%)
Medium SES, 9–14	521 (58.0%)	249 (58.3%)	272 (57.7%)
High SES, 15–21	286 (31.8%)	133 (31.1%)	153 (32.5%)
Puberty status			
Mean Tanner stage, 1–5	2.24 (1.15)	2.57 (1.18)	1.82 (0.95)
1 prepubertal	195 (29.2%)	67 (17.9%)	128 (43.5%)
2 beginning puberty	262 (39.2%)	140 (37.4%)	122 (41.5%)
3 mid‐puberty	104 (15.6%)	84 (22.5%)	20 (6.8%)
4 advanced puberty	69 (10.3%)	51 (13.6%)	18 (6.1%)
5 postpubertal	38 (5.7%)	32 (8.6%)	6 (2.0%)

*Note*: Weight status classification of severe underweight (body mass index standard deviation score, BMI‐SDS ≤‐1.88), underweight (−1.88 < BMI‐SDS ≤ −1.28), normal weight (−1.28 < BMI‐SDS < 1.28), overweight (1.28 ≤ BMI‐SDS < 1.88), and obesity (BMI‐SDS ≥ 1.88).

Abbreviations: ChEDE‐Q, eating disorder examination‐questionnaire for Children, global eating disorder psychopathology; SDQ, Strengths and Difficulties Questionnaire, total behavioral diffic.

^a^ ChEDE‐Q global score without restraint subscale.

### 2.2 Trajectory Variables

Multivariate trajectory analyses were conducted for binge eating, dietary restraint, compensatory behaviors, and BMI‐SDS.

#### 2.2.1 Eating Disorder Examination‐Questionnaire for Children (ChEDE‐Q)

ED symptoms and psychopathology over the past 28 days were assessed using the self‐report version of the Eating Disorder Examination‐Questionnaire for Children (ChEDE‐Q) (Goldschmidt et al. 2007; Hilbert 2016). The ChEDE‐Q comprises 22 items divided into four subscales: restraint, eating concern, weight concern, and shape concern. For each item, frequency or intensity was rated on seven‐point rating scales (0 = *trait was not present* to 6 = *trait was present every day* or *to an extreme degree*). Six additional items assessed the frequency of key ED behaviors over the past 28 days. From the ChEDE‐Q, the following trajectory variables were derived: (1) Binge eating was measured by a single item assessing the frequency of episodes with consumption of a large amount of food accompanied by a feeling of loss of control. (2) Dietary restraint was operationalized using the restraint subscale, calculated as the mean value of its five items, demonstrating good reliability in this sample (McDonald's ω = 0.87). (3) Compensatory behaviors were assessed by summing up the three behavioral items measuring the frequency of vomiting, laxative misuse, and excessive exercise during the past 28 days.

#### 2.2.2 BMI‐SDS

Body mass index (BMI; kg/m^2^) was calculated from objectively measured height and weight and transformed into age‐ and sex‐standardized BMI‐standard deviation scores (BMI‐SDS) using German reference data (Kromeyer‐Hauschild et al. 2001). A BMI‐SDS = 0 corresponds to the 50th percentile, ±1.28 to the 90th/10th percentiles, and ±1.88 to the 97th/3rd percentiles.

### 2.3 Validation and Outcome Measures

Measures for validation and outcome of the trajectory group solution at baseline and last assessment, respectively, included global ED psychopathology and general psychopathology. Additionally, anthropometric and sociodemographic data were assessed for descriptive purposes.

#### 2.3.1 Eating Disorder Psychopathology

ED psychopathology was measured by the global score of the ChEDE‐Q (Goldschmidt et al. 2007; Hilbert 2016), ranging from 0 to 6, with higher scores indicating greater psychopathology. To avoid overlap between trajectory indicators, the restraint subscale was excluded from the ChEDE‐Q global score. This ChEDE‐Q global score demonstrated excellent internal consistency in this study's sample (McDonald's ω = 0.95).

Descriptively, global ED psychopathology was evaluated for clinical significance. Therefore, the proportion of children exceeding the 90th age‐ and sex‐specific percentile of the ChEDE‐Q8 global score was reported for each trajectory (Kliem et al. 2017). The ChEDE‐Q8 was designed so that its global mean score is highly correlated (*r* = 0.97) with that of the ChEDE‐Q (Kliem et al. 2017).

#### 2.3.2 General Psychopathology

The self‐ and parent‐report versions of the Strengths and Difficulties Questionnaire (SDQ; Goodman 1997; Klasen et al. 2003) were used to assess behavioral difficulties. The SDQ consists of 25 items rated on a 3‐point Likert scale (0 = *not true*, 1 = *somewhat true*, and 2 = *certainly true*) across five subscales: emotional symptoms, hyperactivity/inattention, peer relationship problems, conduct problems, and prosocial behavior. The total difficulties sum score, excluding the prosocial behavior subscale, was calculated, ranging from 0 to 40, with higher scores indicating greater difficulties. The total difficulties score showed acceptable internal consistency for the self‐report (McDonald's *ω* = 0.64) and parent‐report version (McDonald's *ω* = 0.67).

To evaluate clinical significance, the proportion of those exceeding the 90th age‐ and sex‐specific percentile based on self‐report (Lohbeck et al. 2015) and parent‐report (Janitza et al. 2020) was evaluated.

#### 2.3.3 Socioeconomic Status (SES)

Participants' socioeconomic status (SES) was assessed using parent reports of their highest level of education, current occupation, and income. Each SES factor was scored on a scale of 1–7 and combined into the Winkler Index, ranging from 3 to 21, with higher scores indicating higher socioeconomic status (Lange et al. 2007). Families were classified as having low (3–8), medium (9–14), or high (15–21) SES.

#### 2.3.4 Anthropometric and Sociodemographic Data

Age and sex of the children and adolescents were assessed by parent‐report. Participants' pubertal stage was assessed by trained research assistants, with Tanner stages ranging from 1 (prepubertal) to 5 (full adult maturity; Tanner 1962).

### 2.4 Data Analytic Plan

Group‐based multi‐trajectory modeling (GBMTM; Nagin et al. 2024; Nagin et al. 2018) was employed to identify latent clusters of children following similar trajectories across the four trajectory variables: binge eating, dietary restraint, compensatory behaviors, and BMI‐SDS. This method extends univariate group‐based trajectory modeling by employing multinomial modeling with maximum likelihood estimation of model parameters under a missing‐at‐random assumption, using all available observations without imputation. Further information on the number of missing data and analyses assessing the plausibility of the missing‐at‐random assumption are reported in the Supplementary Methods and Tables S1–S6. To focus on youth‐specific trajectories and address limited data for participants older than 17.5 years, only time points with participants' age between 9.5 and 17.5 years were included in the analysis. Models ranging from 1 to 7 trajectory groups were estimated, and the optimal model was selected based on the following criteria (Klijn et al. 2017; Lu et al. 2023; Nagin 2005): Bayesian Information Criterion closest to 0, average posterior probability of assignment > 70, odds of correct classification > 5.0, and at least 5% of participants in each group. Details of the model fitting process are provided in the supplement (Supplementary Methods).

The trajectory groups identified through GBMTM were compared with multivariate analysis of variance (MANOVA) to identify group differences at baseline for validation and last available assessment for outcome estimation in ED psychopathology and general psychopathology. Following a significant MANOVA, univariate analyses of variance (ANOVAs) were conducted to examine group differences in validation and outcome measures, with post hoc Games‐Howell‐corrected analyses. GBMTM was performed using Stata's traj package (Jones and Nagin 2013); all other analyses were conducted with IBM SPSS Statistics (Version 29). All statistical tests applied a two‐tailed α < 0.05. As effect size, partial *η* ^2^ and Cramér's *V* were calculated, which can be interpreted as small (*η*_*p*_^2^ ≥ 0.01, *V*[df = 4 or 5] ≥ 0.05), medium (*η*_*p*_^2^ ≥ 0.06, *V*[df = 4] ≥ 0.15, *V*[df = 5] ≥ 0.13), or large (*η*_*p*_^2^ ≥ 0.14, *V*[df = 4] ≥ 0.25, *V*[df = 5] ≥ 0.22; Cohen 1988).

## 3 Results

### 3.1 Girls

**FIGURE 1:**
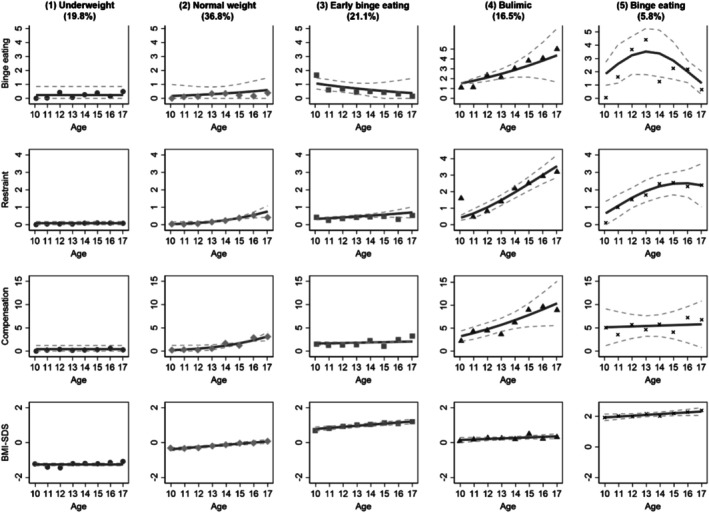
Group‐based multi‐trajectory modeling of binge eating, dietary restraint, compensatory behaviors, and BMI‐SDS in girls (*n* = 427). Binge eating, number of binge‐eating episodes over the past 28 days, Eating Disorder Examination‐Questionnaire for Children (ChEDE‐Q); BMI‐SDS, Body Mass Index Standard Deviation Score; Compensation, sum of compensatory behaviors (vomiting, laxative misuse, excessive exercise) over the past 28 days (ChEDE‐Q); Restraint, Restraint subscale of the ChEDE‐Q, 0–6. Solid lines represent the estimated mean trajectories; dotted lines represent 95% confidence intervals. Percentages in parentheses indicate the proportion of participants assigned to each trajectory group.

For girls, the GBMTM using the four trajectory variables of binge eating, dietary restraint, compensatory behaviors, and BMI‐SDS favored a five‐trajectory solution with mostly linear trajectories (Figure 1, Table S7). Group 1 (19.8%), labeled *underweight*, showed persistently low ED symptoms and a stable, low BMI‐SDS. Group 2 (36.8%), labeled *normal weight*, showed slight increases in binge eating, restraint, and compensatory behaviors at low levels, together with a small increase in BMI‐SDS within the normal‐weight range. Group 3 (21.1%), labeled *early binge eating*, presented with initially slightly elevated but decreasing binge eating, low restraint and compensatory behaviors, and an increase in BMI‐SDS into the higher normal‐weight range. Group 4 (16.5%), labeled *bulimic*, showed increases in binge eating, compensatory behaviors, and restraint while maintaining a stable normal‐weight BMI‐SDS. Group 5 (5.8%), labeled *binge eating*, showed increasing early binge eating followed by decreases, moderate dietary restraint and compensatory behaviors, and a slightly increasing BMI‐SDS within the obesity range.

#### 3.1.1 Validation

Following a significant multivariate effect, *p* < 0.001, univariate analyses indicated significant differences in ED psychopathology (large effect) and self‐ and parent‐reported general psychopathology (small effects; Table 2) across trajectories. Post hoc tests showed higher levels of ED psychopathology in the *binge eating* group than in all other groups. The *bulimic* and *early binge eating* groups were characterized by higher ED psychopathology than the *underweight* and *normal weight* groups. Concerning parent‐reported general psychopathology, the *binge eating* group showed more difficulties than the *underweight* and the *normal weight* groups, with 36% exceeding the 90th percentile in the SDQ (Table S9).

**TABLE 2:** Validation of trajectory groups identified through group‐based multi‐trajectory modeling in girls at baseline (*n* = 427).

Variable	(1) Underweight		(2) Normal weight		(3) Early binge eating		(4) Bulimic		(5) Binge eating		*F*	df	*p*	*η* ^2^ (95% CI)
	*M* (SD)/*n* (%)	*n*	*M* (SD)/*n* (%)	*n*	*M* (SD)*/n* (%)	*n*	*M* (SD)*/n* (%)	*n*	*M* (SD)*/n* (%)	*n*				
Sociodemographics														
Age, years	11.94 (1.52)	83	11.81 (1.47)	160	11.67 (1.31)	90	11.92 (1.48)	69	11.96 (1.56)	25	0.51	4, 422	0.726	0.01 (0.00–0.02)
Puberty status														
Mean tanner stage	2.29 (1.11)^A^	70	2.49 (1.22)^AB^	140	2.90 (1.15)^B^	80	2.60 (1.09)^AB^	62	2.82 (1.26)^AB^	22	3.08	4, 369	0.016	0.03 (0.00–0.07)
1. Prepubertal	18 (25.7%)		34 (24.3%)		5 (6.3%)		8 (12.9%)		2 (9.1%)					
2. Beginning puberty	28 (40.0%)		46 (32.9%)		30 (37.5%)		27 (43.6%)		9 (40.9%)					
3. Mid‐puberty	13 (18.6%)		28 (20.0%)		25 (31.3%)		12 (19.4%)		6 (27.3%)					
4. Advanced puberty	8 (11.4%)		22 (15.7%)		8 (10.0%)		12 (19.4%)		1 (4.6%)					
5. Postpubertal	3 (4.3%)		10 (7.1%)		12 (15.0%)		3 (4.8%)		4 (18.2%)					
SES														
Mean Winkler index	14.10 (3.28)^A^	80	13.99 (3.21)^A^	149	13.42 (3.34)^A^	88	14.25 (3.38)^A^	69	12.03 (3.78)^A^	24	2.60	4, 405	0.036	0.03 (0.00–0.05)
Low SES	3 (3.8%)		10 (6.7%)		7 (8.0%)		4 (5.8%)		4 (16.7%)					
Medium SES	48 (60.0%)		91 (61.1%)		55 (62.5%)		38 (55.1%)		17 (70.8%)					
High SES	29 (36.3%)		48 (32.2%)		26 (29.6%)		27 (39.1%)		3 (12.5%)					
Trajectory indicators											32.88	16, 1660.00	< 0.001	
Binge eating	0.27 (1.14)^A^	83	0.23 (0.98)^A^	157	0.81 (1.83)^AB^	89	1.87 (4.14)^B^	69	1.96 (3.11)^AB^	24	5.92	4, 108.25	< 0.001	0.09 (0.04–0.13)
Restraint	0.04 (0.13)^A^	83	0.12 (0.30)^B^	160	0.38 (0.70)^C^	90	1.08 (1.48)^D^	69	1.72 (1.40)^D^	25	22.73	4, 112.35	< 0.001	0.28 (0.21–0.34)
Compensation	0.27 (0.91)^A^	83	0.64 (2.86)^AB^	157	2.18 (5.52)^BC^	89	4.97 (7.11)^C^	69	5.04 (6.46)^C^	24	12.99	4, 109.09	< 0.001	0.14 (0.08–0.19)
BMI‐SDS	−1.41 (0.63)^A^	82	−0.31 (0.47)^B^	160	0.90 (0.45)^C^	89	0.15 (0.53)^D^	69	1.94 (0.42)^E^	25	333.93	4, 420	< 0.001	0.76 (0.72–0.79)
Validation variables											13.76	12, 1173.00	< 0.001	
ChEDE‐Q^a^	0.26 (0.38)^A^	83	0.34 (0.50)^A^	160	1.03 (1.11)^B^	90	1.32 (1.33)^B^	69	2.45 (1.27)^C^	25	34.11	4, 113.82	< 0.001	0.32 (0.24–0.38)
SDQ self‐report	10.79 (5.34)^A^	80	9.42 (4.25)^A^	156	10.31 (4.82)^A^	87	10.95 (5.49)^A^	64	12.64 (5.04)^A^	22	3.07	4, 108.51	0.019	0.03 (0.00–0.06)
SDQ parent‐report	7.28 (4.61)^A^	78	6.70 (4.92)^A^	153	7.84 (5.33)^AB^	88	8.01 (5.03)^AB^	68	11.18 (5.97)^B^	25	4.61	4, 407	0.001	0.04 (0.01–0.08)

*Note*: Binge eating, number of binge‐eating episodes over the past 28 days, Eating Disorder Examination‐Questionnaire for Children (ChEDE‐Q); BMI‐SDS, Body Mass Index Standard Deviation Score; Compensation, sum of compensatory behaviors (vomiting, laxative misuse, excessive exercise) over the past 28 days (ChEDE‐Q); Restraint, Restraint subscale of the ChEDE‐Q, 0–6; ChEDE‐Q, Eating Disorder Examination‐Questionnaire for Children, global eating disorder psychopathology, 0–6; Puberty status, Tanner stage, 1–5; SDQ, Strengths and Difficulties Questionnaire, total behavioral difficulties, 0–40; SES, Socioeconomic status, Winkler index, 3–21. For puberty status and socio‐economic status (SES), the first row presents the mean (SD), and the subsequent rows present the distribution across categories as *n* (column %). ANOVA assessed group differences in age and puberty. MANOVA assessed overall group differences in ChEDE‐Q and SDQ, with follow‐up ANOVAs (Welch ANOVA applied for violations of homogeneity of variances). ^A,B,C,D,E^Different superscripts denote significant post hoc differences (Games‐Howell). *p* < 0.05, two‐tailed.

^a^ ChEDE‐Q global score without Restraint subscale.

#### 3.1.2 Outcome

Based on a significant multivariate effect, *p* < 0.001, univariate analyses revealed significant differences in ED psychopathology (large effect) and self‐ and parent‐reported general psychopathology (medium effects) at last assessment between the trajectories (Table 3). Post hoc tests indicated that the *binge eating* and *bulimic* groups had higher levels of ED psychopathology than all other groups. In addition, the *early binge eating* group showed higher ED psychopathology than the *normal weight* and the *underweight* groups, which also differed significantly from one another. The *normal weight* group was characterized by lower general psychopathology than the *bulimic* and *binge eating* groups on both self‐ and parent‐report. Participants in the *early binge eating* group showed lower general psychopathology than the *bulimic* group based on self‐report and relative to the *binge eating* group based on parent‐report.

**TABLE 3:** Indicator and outcome variables of trajectory groups identified through group‐based multi‐trajectory modeling at last assessment in girls (*n* = 427).

Variable	(1) Underweight		(2) Normal weight		(3) Early binge eating		(4) Bulimic		(5) Binge eating		*F*	df	*p*	*η* ^2^ (95% CI)
	*M* (SD)/*n* (%)	n	*M* (SD)/*n* (%)	n	*M* (SD)/*n* (%)	n	*M* (SD)/*n* (%)	*n*	*M* (SD)/*n* (%)	*n*				
Trajectory indicators											40.98	16, 1672.00	< 0.001	
Binge eating	0.36 (1.45)^A^	83	0.26 (0.88)^A^	160	0.28 (0.79)^A^	90	3.41 (6.77)^B^	69	3.44 (5.56)^AB^	25	5.67	4, 111.28	< 0.001	0.15 (0.08–0.20)
Restraint	0.09 (0.23)^A^	83	0.34 (0.71)^B^	160	0.44 (0.54)^B^	90	2.11 (1.81)^C^	69	2.04 (1.77)^C^	25	35.73	4, 114.59	< 0.001	0.37 (0.30–0.43)
Compensation	0.43 (1.35)^A^	83	1.34 (3.59)^B^	160	1.91 (4.76)^B^	90	6.94 (8.46)^C^	69	4.56 (6.51)^BC^	25	14.56	4, 114.14	< 0.001	0.17 (0.10–0.23)
BMI‐SDS	−1.21 (0.55)^A^	83	−0.05 (0.48)^B^	159	1.10 (0.48)^C^	90	0.23 (0.62)^D^	67	2.27 (0.61)^E^	25	315.93	4, 418	< 0.001	0.75 (0.71–0.78)
Outcome variables											18.42	12, 1209.00	< 0.001	
ChEDE‐Q^a^	0.32 (0.42)^A^	83	0.68 (0.86)^B^	160	1.13 (0.93)^C^	90	2.54 (1.46)^D^	69	2.92 (1.37)^D^	25	63.89	4, 116.99	< 0.001	0.43 (0.36–0.49)
SDQ self‐report	10.28 (4.73)^AB^	83	9.16 (5.03)^A^	159	9.36 (4.73)^AC^	90	12.33 (6.11)^B^	69	13.24 (6.27)^BC^	25	7.29	4, 421	< 0.001	0.07 (0.02–0.11)
SDQ parent‐report	7.18 (4.93)^AB^	79	6.18 (4.52)^A^	149	6.68 (4.74)^AC^	87	9.04 (5.86)^BC^	68	10.88 (6.41)^B^	25	5.61	4, 117.79	< 0.001	0.07 (0.02–0.11)

*Note*: Binge eating, number of binge‐eating episodes over the past 28 days, Eating Disorder Examination‐Questionnaire for Children (ChEDE‐Q); BMI‐SDS, Body Mass Index Standard Deviation Score; Compensation, sum of compensatory behaviors (vomiting, laxative misuse, excessive exercise) over the past 28 days (ChEDE‐Q); Restraint, Restraint subscale of the ChEDE‐Q, 0–6; ChEDE‐Q, Eating Disorder Examination‐Questionnaire for Children, global eating disorder psychopathology, 0–6; SDQ, Strengths and Difficulties Questionnaire, total behavioral difficulties, 0–40. MANOVA assessed overall group differences, with follow‐up ANOVAs (Welch ANOVA applied for violations of homogeneity of variances). ^A,B,C,D,E^Different superscripts denote significant post hoc differences (Games‐Howell). *p* < 0.05, two‐tailed.

^a^ ChEDE‐Q global score without Restraint subscale.

Regarding clinical significance, more than 20% and 32% of children in the *bulimic* and *binge eating* groups exceeded the 90th percentile on the SDQ by self‐ and parent‐report at last assessment (Table S9). On the ChEDE‐Q, while no participant in the *underweight, normal weight*, and the *early binge eating* groups scored above the 90th percentile at last assessment, 18.8% and 24.0% of participants in the *bulimic* and *binge eating* groups showed clinically significant ED psychopathology.

### 3.2 Boys

**FIGURE 2:**
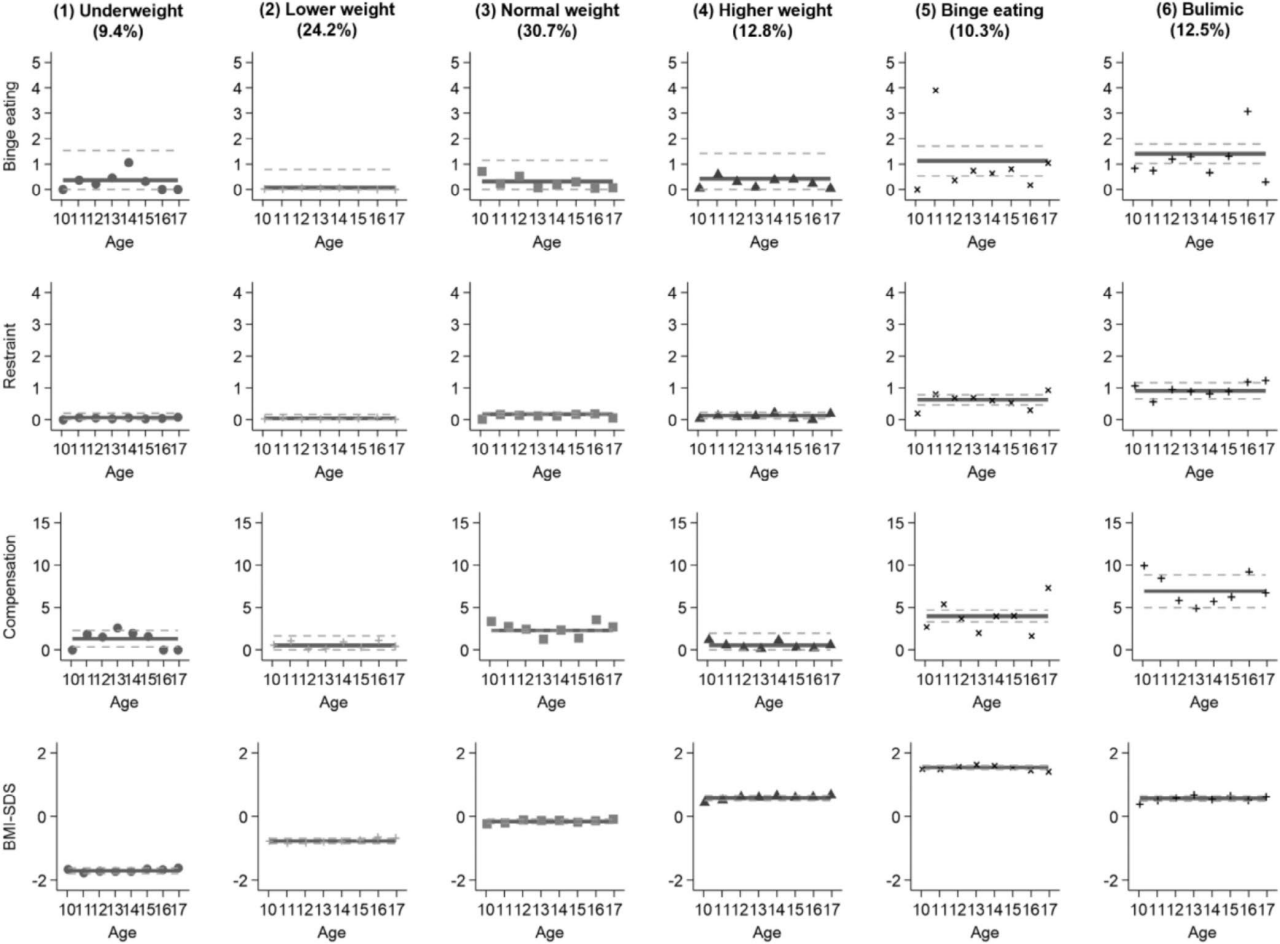
Group‐based multi‐trajectory modeling of binge eating, dietary restraint, compensatory behaviors, and BMI‐SDS in boys (*n* = 471). Binge eating, number of binge‐eating episodes over the past 28 days, Eating Disorder Examination‐Questionnaire for Children (ChEDE‐Q); BMI‐SDS, Body Mass Index Standard Deviation Score; Compensation, sum of compensatory behaviors (vomiting, laxative misuse, excessive exercise) over the past 28 days (ChEDE‐Q); Restraint, Restraint subscale of the ChEDE‐Q, 0–6. Solid lines represent the estimated mean trajectories; dotted lines represent 95% confidence intervals. Percentages in parentheses indicate the proportion of participants assigned to each trajectory group.

For boys, model selection favored a six‐group solution (Table S8). As presented in Figure 2, the six trajectories in boys showed largely stable patterns in ED symptoms and BMI‐SDS over time. Group 1 (9.4%), labeled *underweight*, showed low binge eating, restraint, and compensatory behaviors together with a persistently low BMI‐SDS. Group 2 (24.2%), labeled *lower weight*, was characterized by virtually absent ED symptoms and stable lower normal‐weight BMI‐SDS. Group 3 (30.7%), labeled *normal weight*, showed low but stable binge eating and slightly increased compensatory behaviors at otherwise low restraint and stable normal‐weight BMI‐SDS. Group 4 (12.8%), labeled *higher weight*, showed persistently low ED symptoms and a higher, stable normal‐weight BMI‐SDS. Group 5 (10.3%), labeled *binge eating*, exhibited elevated binge eating and restraint, moderate and stable compensatory behaviors, and a persistently high BMI‐SDS in the obesity range. Group 6 (12.5%), labeled *bulimic*, showed moderate binge eating and restraint and high and stable compensatory behaviors at a higher normal‐weight BMI‐SDS.

#### 3.2.1 Validation

Based on a significant multivariate effect, *p* < 0.001, follow‐up ANOVAs revealed significant group differences in global ED psychopathology (large effects), while no differences were seen for self‐ and parent‐reported general psychopathology (Table 4). Boys in the *binge eating* and *bulimic* groups reported higher levels of ED psychopathology than all other groups, which did not differ from each other.

**TABLE 4:** Validation of trajectory groups identified through group‐based multi‐trajectory modeling in boys at baseline (*n* = 471).

Variable	(1) Underweight		(2) Lower weight		(3) Normal weight		(4) Higher weight		(5) Binge eating		(6) Bulimic		*F*	df	*p*	η^2^ (95% CI)
	*M* (SD)/*n* (%)	*n*	*M* (SD)/*n* (%)	*n*	*M* (SD)/*n* (%)	*n*	*M* (SD)/*n* (%)	*n*	*M* (SD)/*n* (%)	*n*	*M* (SD)/*n* (%)	*n*				
Sociodemographics																
Age, years	11.61 (1.07)	45	11.76 (1.38)	113	11.88 (1.49)	145	11.47 (1.12)	61	11.97 (1.35)	48	12.19 (1.62)	59	2.23	5, 170.85	0.053	0.02 (0.00–0.04)
Puberty status																
Mean tanner stage	1.48 (0.58)^A^	27	1.71 (0.89)^AB^	77	1.74 (0.83)^AB^	82	1.98 (1.00)^AB^	42	2.29 (1.27)^B^	28	1.89 (1.09)^AB^	38	2.67	5, 102.80	0.026	0.04 (0.00–0.08)
1. Prepubertal	15 (55.6%)		36 (46.8%)		36 (43.9%)		15 (35.7%)		8 (28.6%)		18 (47.5%)					
2. Beginning puberty	11 (40.7%)		34 (44.2%)		36 (43.9%)		18 (42.9%)		12 (42.9%)		11 (29.0%)					
3. Mid‐puberty	1 (3.7%)		1 (1.3%)		5 (6.1%)		5 (11.9%)		3 (10.7%)		5 (13.2%)					
4. Advanced puberty	0 (0%)		5 (6.5%)		5 (6.1%)		3 (7.1%)		2 (7.1%)		3 (7.9%)					
5. Postpubertal	0 (0%)		1 (1.3%)		0 (0%)		1 (2.4%)		3 (10.7%)		1 (2.6%)					
SES																
Mean Winkler index	14.34 (3.28)	44	14.18 (3.04)	111	14.07 (3.19)	136	14.04 (3.24)	60	13.04 (3.22)	45	13.70 (3.51)	57	1.08	5, 447	0.371	0.01 (0.00–0.03)
Low SES	4 (4.6%)		14 (5.6%)		10 (6.0%)		12 (12.5%)		7 (4.5%)		9 (8.5%)					
Medium SES	52 (59.8%)		148 (59.2%)		102 (60.7%)		66 (68.8%)		94 (60.3%)		59 (55.7%)					
High SES	31 (35.6%)		88 (35.2%)		56 (33.3%)		18 (18.8%)		55 (35.3%)		38 (35.9%)					
Trajectory indicators													31.46	20, 1804.00	< 0.001	
Binge eating	0.24 (1.23)^AB^	45	0.02 (0.13)^A^	110	0.25 (0.92)^B^	142	0.52 (1.71)^AB^	60	2.33 (9.42)^AB^	43	1.25 (2.68)^B^	59	5.85	5, 133.97	< 0.001	0.05 (0.01–0.08)
Restraint	0.07 (0.21)^A^	45	0.05 (0.22)^A^	113	0.13 (0.30)^A^	145	0.14 (0.45)^A^	61	0.87 (1.42)^B^	48	0.83 (0.95)^B^	59	11.32	5, 157.89	< 0.001	0.21 (0.14–0.26)
Compensation	1.44 (4.54)^AB^	45	0.82 (3.65)^A^	110	2.85 (6.46)^B^	142	0.42 (1.47)^A^	60	4.23 (6.91)^BC^	44	8.56 (9.03)^C^	59	14.29	5, 158.42	< 0.001	0.16 (0.10–0.21)
BMI‐SDS	−1.66 (0.47)^A^	45	−0.81 (0.35)^B^	113	−0.19 (0.35)^C^	145	0.49 (0.36)^D^	60	1.52 (0.35)^E^	48	0.59 (0.43)^D^	59	485.39	5, 464	< 0.001	0.84 (0.82–0.86)
Validation variables													7.47	15, 1296.00	< 0.001	
ChEDE‐Q^a^	0.15 (0.25)^A^	45	0.17 (0.32)^A^	113	0.25 (0.36)^A^	145	0.34 (0.58)^A^	61	0.98 (1.18)^B^	48	1.07 (1.18)^B^	59	12.02	5, 160.09	< 0.001	0.22 (0.15–0.27)
SDQ self‐report	9.41 (6.40)	44	9.71 (5.44)	105	9.17 (4.62)	140	10.24 (4.64)	59	10.33 (5.41)	48	11.01 (4.67)	58	1.33	5, 172.95	0.251	0.02 (0.00–0.03)
SDQ parent‐report	9.91 (7.37)	44	8.46 (5.84)	111	8.14 (5.00)	138	8.84 (5.56)	58	8.58 (5.96)	45	9.23 (6.80)	58	0.76	5, 448	0.581	0.01 (0.00–0.02)

*Note*: Binge eating, number of binge‐eating episodes over the past 28 days, Eating Disorder Examination‐Questionnaire for Children (ChEDE‐Q); BMI‐SDS, Body Mass Index Standard Deviation Score; Compensation, sum of compensatory behaviors (vomiting, laxative misuse, excessive exercise) over the past 28 days (ChEDE‐Q); Restraint, Restraint subscale of the ChEDE‐Q, 0–6; ChEDE‐Q, Eating Disorder Examination‐Questionnaire for Children, global eating disorder psychopathology, 0–6; Puberty status, Tanner stage, 1–5; SDQ, Strengths and Difficulties Questionnaire, total behavioral difficulties, 0–40; SES, Socioeconomic status, Winkler index, 3–21. For puberty status and socioeconomic status (SES), the first row presents the mean (SD), and the subsequent rows present the distribution across categories as *n* (column %). ANOVA assessed group differences in age and puberty. MANOVA assessed overall group differences in ChEDE‐Q and SDQ, with follow‐up ANOVAs (Welch ANOVA applied for violations of homogeneity of variances). ^A,B,C,D,E^Different superscripts denote significant post hoc differences (Games‐Howell). *p* < 0.05, two‐tailed.

^a^ ChEDE‐Q global score without Restraint subscale.

#### 3.2.2 Outcomes

Given a significant multivariate effect, *p* < 0.001, univariate analyses showed significant differences between trajectory groups in ED psychopathology (large effect), while no effects were seen for self‐ and parent‐reported general psychopathology (small effects, Table 5). Boys in the *binge eating* and *bulimic* groups reported higher ED psychopathology than all other groups. In contrast, the *underweight*, *lower weight*, *normal weight, and higher weight* groups showed mostly consistently low ED psychopathology.

**TABLE 5:** Indicator and outcome variables of trajectory groups identified through group‐based multi‐trajectory modeling at last assessment in boys (*n* = 471).

Variable	(1) Underweight		(2) Lower weight		(3) Normal weight		(4) Higher weight		(5) Binge eating		(6) Bulimic		*F*	df	*p*	*η* ^2^ (95% CI)
	*M* (SD)/*n* (%)	*n*	*M* (SD)/*n* (%)	*n*	*M* (SD)*/n* (%)	*n*	*M* (SD)/*n* (%)	*n*	*M* (SD)/*n* (%)	*n*	*M* (SD)/*n* (%)	*n*				
Trajectory indicators													32.86	20, 1860	< 0.001	
Binge eating	0.82 (3.26)^A^	45	0.05 (0.35)^A^	113	0.14 (1.12)^A^	145	0.51 (2.10)^A^	61	0.73 (1.84)^A^	48	0.93 (3.10)^A^	59	3.21	5, 141.81	0.009	0.03 (0.00–0.06)
Restraint	0.03 (0.13)^AB^	45	0.03 (0.11)^A^	113	0.12 (0.37)^B^	145	0.06 (0.18)^AB^	61	0.58 (0.82)^C^	48	0.91 (1.03)^C^	59	14.16	5, 160.11	< 0.001	0.27 (0.20–0.32)
Compensation	1.82 (6.59)^A^	45	0.72 (3.12)^A^	113	1.64 (3.87)^A^	145	0.52 (3.15)^A^	61	3.10 (6.24)^AB^	48	6.15 (7.20)^B^	59	7.85	5, 157.08	< 0.001	0.12 (0.06–0.17)
BMI‐SDS	−1.73 (0.50)^A^	45	−0.75 (0.43)^B^	113	−0.14 (0.42)^C^	145	0.69 (0.37)^D^	61	1.64 (0.44)^E^	48	0.60 (0.42)^D^	59	415.27	5, 465	< 0.001	0.82 (0.79–0.84)
Outcome variables													8.38	15, 1290.00	< 0.001	
ChEDE‐Q^a^	0.20 (0.30)^AB^	45	0.12 (0.19)^A^	113	0.23 (0.38)^B^	145	0.15 (0.25)^AB^	61	0.73 (0.81)^C^	48	0.94 (0.93)^C^	59	14.96	5, 156.08	< 0.001	0.26 (0.19–0.31)
SDQ self‐report	8.22 (4.70)	45	7.92 (4.01)	113	8.51 (5.12)	144	8.72 (5.33)	61	9.64 (6.26)	47	9.44 (5.06)	59	1.22	5, 162.14	0.302	0.01 (0.00–0.03)
SDQ parent‐report	7.64 (5.46)	42	6.84 (4.84)	108	7.31 (5.38)	140	7.53 (4.56)	57	8.28 (4.82)	39	7.49 (5.82)	51	0.51	5, 431	0.768	0.01 (0.00–0.02)

*Note*: Binge eating, number of binge‐eating episodes over the past 28 days, Eating Disorder Examination‐Questionnaire for Children (ChEDE‐Q); BMI‐SDS, Body Mass Index Standard Deviation Score; Compensation, sum of compensatory behaviors (vomiting, laxative misuse, excessive exercise) over the past 28 days (ChEDE‐Q); Restraint, Restraint subscale of the ChEDE‐Q, 0–6; ChEDE‐Q, Eating Disorder Examination‐Questionnaire for Children, global eating disorder psychopathology, 0–6; Puberty status, Tanner stage, 1–5; SDQ, Strengths and Difficulties Questionnaire, total behavioral difficulties, 0–40. MANOVA assessed overall group differences, with follow‐up ANOVAs (Welch ANOVA applied for violations of homogeneity of variances). ^A,B,C,D,E^ Different superscripts denote significant post hoc differences (Games‐Howell). *p* < 0.05, two‐tailed.

^a^ ChEDE‐Q global score without Restraint subscale.

Descriptively, a small proportion (3.4%) of boys in the *bulimic* group scored above the 90th percentile on global ED psychopathology at last assessment (Table S10). No other trajectory groups presented with clinically significant ED psychopathology.

## 4 Discussion

In a large longitudinal community‐based cohort of youth aged 9.5–17.5 years, followed for an average of 3 years (range 2–6 years), multivariate person‐centered latent structure analysis of core behavioral ED symptoms and age‐adjusted BMI using GBMTM identified five distinct trajectories in girls and six in boys. By categorizing adolescents based on joint changes in binge eating, restraint, compensatory behaviors, and age‐adjusted BMI, two at‐risk trajectories were identified in each sex: *bulimic* (16.5% of girls, 12.5% of boys) and *binge eating* (5.8% of girls, 10.3% of boys), beyond low‐symptom trajectories with underweight and normal‐weight profiles and, in girls, an intermediate‐risk group with *early binge eating* (21.1%) at higher normal‐weight BMI (Table S11). These findings align with known prevalence and relative stability of ED symptoms in adolescence (Foster and Daukantaité 2024; López‐Gil et al. 2023) and associations with sex and puberty (Bould et al. 2017; Rolan et al. 2023), while extending prior univariate trajectory modeling (e.g., Mattsson et al. 2019; Pearson and Smith 2015; Smith et al. 2007) by delineating multivariate patterns of binge‐eating‐related symptoms and weight status across adolescence.

The majority of girls and boys followed stable, low‐symptom pathways in which lower or normal weight co‐occurred with persistently low or only slightly increasing levels of binge eating, compensatory behaviors, and restraint, suggesting that modest fluctuations in single symptoms and weight‐related changes are often developmentally normative and not sufficient markers of clinically relevant ED psychopathology. In girls, the *underweight* and *normal weight* trajectories accounted for 56.6% of the sample, consistent with previous studies showing that the majority of adolescent girls in community samples followed asymptomatic or low‐symptom courses (e.g., Breton et al. 2022; Pearson and Smith 2015; Verschueren et al. 2020). The developmentally normative low increase in ED symptoms during adolescence has been previously documented, especially in girls (Ackard et al. 2011). In boys, four low‐symptom trajectories across the weight range together encompassed 77.1% of participants, in line with findings by Verschueren et al. (2020), who identified a latent developmental trajectory with virtually absent ED symptoms at normal weight in 82% of adolescent boys. The fact that these low‐symptom groups did not differ in general psychopathology at either baseline or last assessment supports the validity of these trajectory groups. Distinguishing various low‐symptom trajectories is informative, as it shows that lower, normal, and higher age‐adjusted BMI can each co‐occur with persistently low ED symptoms across adolescence.

Beyond these low‐symptom trajectories, GBMTM revealed an intermediate‐risk group of girls characterized by *early binge eating*. In this group, binge eating decreased over adolescence, while BMI‐SDS continuously increased into the higher normal‐weight range. At baseline, 4.4% and 13.6% of girls in this trajectory exceeded the cutoff for clinically relevant ED and parent‐reported psychopathology; parent‐reported psychopathology did not differ from the *bulimic* and *binge eating* trajectories at this time. However, in contrast to the *bulimic* and *binge eating* trajectories, ED symptoms and general psychopathology in the *early binge eating* group remained stable or slightly decreased over time. A similar divergence between decreasing and increasing binge‐eating trajectories has been reported by Pearson and Smith (2015), who observed higher ED psychopathology at baseline in girls with decreasing (early onset) binge eating compared to those with increasing binge eating, and higher ED psychopathology in the increasing binge‐eating group by late adolescence. Taken together, this pattern points to a subgroup of young girls in whom ED‐related behaviors may emerge transiently in the context of higher (normal) weight without consolidating into persistent psychopathology, consistent with evidence that most adolescents with overweight do not develop clinically significant EDs (Loth et al. 2015; Schlüter et al. 2016; Zeiler et al. 2021).

From a developmental perspective, the most salient findings concern the high‐risk trajectories characterized by persistently elevated or increasing ED symptoms at normal or higher weight. In girls, the *bulimic* trajectory was characterized by increasing binge eating, restraint, and compensatory behaviors in later adolescence, alongside relatively stable normal‐weight age‐adjusted BMI, consistent with evidence on bulimia nervosa onset in late adolescent females or young women (Micali et al. 2015; Stice 2016; Stice, Desjardins, et al. 2021). The *binge eating* trajectory showed early increases in binge eating and persistently high age‐adjusted BMI, in line with reports of an early peak onset of binge‐eating disorder in adolescent girls (Stice et al. 2013). The considerable number of compensatory behaviors, mainly excessive exercise, which is often overreported (Binford et al. 2005; Reas et al. 2011), in the *binge eating* trajectory is consistent with findings that adolescents with binge‐eating disorder may display such behaviors before the onset of regular binge eating, potentially “trying out” compensatory weight management (Davis et al. 2018; Yamamiya et al. 2023). Despite their different developmental timing and weight profiles, both trajectories converged on similarly elevated levels of ED psychopathology at last assessment, suggesting that different developmental routes lead to comparable clinical burden. Although post hoc comparisons were not significant, girls in the *binge eating* trajectory had the highest proportion of families with low socioeconomic status (16.7%) compared to all other trajectory groups (≤ 8.0%), in line with findings that low SES in adolescence predicts later obesity (Poulsen et al. 2018; Sares‐Jäske et al. 2022).

In boys, two high‐risk trajectories, representing 23% of the sample, were characterized by relatively low binge eating but pronounced and persistent compensatory behaviors at normal and higher weight. This pattern is consistent with evidence that boys are more likely to endorse excessive exercise as a compensatory behavior than girls while underreporting binge‐eating symptoms (Parker et al. 2023). The *binge eating* and *bulimic* trajectory groups were largely indistinguishable in ED and general psychopathology, differing mainly in BMI‐SDS, both at baseline and last assessment. Notably, the *bulimic* trajectory was the only group in which a small proportion of boys (3.4%) exceeded the cutoff for clinically relevant ED psychopathology at last assessment—a subgroup that may warrant particular attention in early identification efforts.

Overall, symptom levels for binge eating and restraint were lower in boys than girls, underscoring typical gender differences in ED development (López‐Gil et al. 2023). Only in girls the high‐risk trajectories revealed higher general psychopathology than the other groups, consistent with findings supporting a closer association of ED symptoms such as binge eating and dieting with symptoms of other mental disorders in females (Davison et al. 2014). Including sex‐specific ED symptoms, such as muscle‐building, eating more, or night eating in boys (Mitchison et al. 2020; Nagata et al. 2020), is likely to specify trajectory analysis results.

In contrast to the *bulimic* and *binge‐eating* trajectories identified in this study, there was no distinct anorexia nervosa (AN) trajectory, which likely reflects the low base rate of AN in adolescence (Mitchison et al. 2020; Swanson et al. 2011) and the community‐based recruitment (Poulain et al. 2017). Few participants had underweight, and annual assessments may have missed shorter episodes of clinically significant weight loss. Moreover, the trajectory indicators included binge eating, compensatory behaviors, restraint, and age‐adjusted BMI; considering additional AN‐specific indicators (e.g., fear of weight gain, shape concern) may allow finer differentiation of restrictive and atypical AN trajectories in future work.

Strengths of this study include the large prospective sample with a broad age range and sociodemographically comparable subsamples of girls and boys, and the use of well‐established assessments, including objectively measured BMI and validated measures of ED symptoms as well as ED and general psychopathology. Limitations involve self‐reported ED symptoms, which may lead to biases (e.g., underreporting of binge eating; Lange et al. 2025; Reas et al. 2011), and the absence of race/ethnicity and gender identity data. Although the study covered an age range from 9 to 17 years, relatively few adolescents contributed data at the upper end of this range, with a mean age of 14.5 years at the last assessment. Thus, estimates for later ages are based on fewer observations and should be interpreted with caution. The sample was community‐based and unweighted rather than probability‐sampled, which may limit generalizability to the broader German population. Different compensatory behaviors (e.g., self‐induced vomiting, excessive exercise) were combined into a single indicator based on conceptual similarity and low base rates of each behavior, precluding separate analyses. However, this aggregation may have obscured potentially distinct trajectories for specific compensatory behaviors, which should be examined in future studies. Further, the SDQ total difficulties score showed only moderate internal consistency in our sample, which is well‐documented in the literature (e.g., Essau et al. 2012), but may limit the precision with which general psychopathology was captured. As with any exploratory person‐centered longitudinal latent structure approach, the results are an approximation of ‘true’ trajectory classes but do not represent them, with certain simplifications being inherent (Elsenburg et al. 2024).

In conclusion, this study highlights sex‐specific, developmentally distinct trajectories of ED symptoms and weight status across adolescence. From a research perspective, longer follow‐up into young adulthood and interview‐based ED diagnoses are needed to clarify the prognostic significance of the identified trajectories for the onset and persistence of EDs. Clinically, early identification of high‐risk youth may benefit from multi‐symptom tracking rather than a sole focus on BMI or binge eating, with particular attention to emerging bulimic or early binge‐eating symptoms in girls and persistent compensatory behaviors in boys. Incorporating these multivariate and sex‐specific patterns into prevention and early intervention programs (Stice, Desjardins, et al. 2021; Stice, Onipede, and Marti 2021) may help to interrupt high‐risk developmental courses before they consolidate into full‐threshold EDs, for example, by integrating ED prevention components into weight‐management programs for youth with higher weight and by explicitly targeting unhealthy weight‐control behaviors (e.g., excessive exercise) in interventions for boys.

## Author Contributions

Conception and design: Anja Hilbert. Acquisition of data: Andreas Hiemisch, Antje Körner, and Wieland Kiess. Analysis of data: Danielle Schewe, Ricarda Schmidt. Drafting the manuscript: Anja Hilbert and Danielle Schewe. Revising the manuscript critically for important content and final approval of the manuscript and agreement to be accountable: All authors.

## Funding

This publication is supported by the Leipzig Research Center for Civilization Diseases (LIFE), Leipzig University, funded by the European Union, the European Social Fund (ESF), the European Regional Development Fund (ERDF), and the Free State of Saxony within the framework of the excellence initiative. The funding source had no role in study design, data collection, data analysis, reporting of this study, and submission for publication.

## Disclosure

During the preparation of this work the authors used ChatGPT (OpenAI, version 5.0 and 5.1) and DeepL (DeepL SE) in order to improve the readability and language of the manuscript. After using this tool, the authors reviewed and edited the content as needed and took full responsibility for the content of the published article.

## Ethics Statement

Ethical approval was granted by the Ethics Committee of the Medical Faculty of Leipzig University (Ref. No. 264/10‐ek). Informed written consent was obtained at the outset of the study.

## Conflicts of Interest

Dr. Hilbert reports receiving research grants from the German Federal Ministry of Education and Research, German Research Foundation, Innovation Fund, and Roland Ernst Foundation for Health Care; royalties for books on the treatment of eating disorders and obesity with Hogrefe and Kohlhammer; honoraria for workshops and lectures on eating disorders and obesity and their treatment, including from Lilly, Novo Nordisk, and Rhythm Pharmaceuticals; honoraria as editor of the *International Journal of Eating Disorders*; and honoraria as a consultant for Takeda and Medice. No other conflicts of interest were reported.

## Supporting information


**Data S1:** eat70045‐sup‐0001‐Supinfo.docx.

## Data Availability

The data that support the findings of this study are available from the corresponding author upon reasonable request.
