# Supplementary material for: Multivariate Trajectories of Eating Disorder Symptoms and Weight Status in 10‐ to 17‐Year‐Old Children and Adolescents
Source: Int J Eat Disord. 2026 Feb 6;59(5):978–95. doi: 10.1002/eat.70045 (PMC13147146; doi:10.1002/eat.70045)
Supplement: Supplementary file 1 — Data S1: eat70045‐sup‐0001‐Supinfo.docx. [file EAT-59-978-s001.docx]

**Online Supplementary Material for:**

**Multivariate trajectories of eating disorder symptoms and weight status in 10- to 17-year-old children and adolescents**

Anja Hilbert, Danielle Schewe, Andreas Hiemisch, Antje Körner, Wieland Kiess, Ricarda Schmidt

**Supplementary Method**

**Supplementary Results**

**Supplementary References**

**Supplementary Tables**

**Supplementary Table S1.** Number of eligible, observed, and missing values for each indicator variable at each age in girls (*n*=427).

**Supplementary Table S2.** Number of eligible, observed, and missing values for each indicator variable at each age in boys (*n*=471).

**Supplementary Table S3.** Fit indices for group-based multi-trajectory modeling in girls (*n*=427).

**Supplementary Table S4.** Fit indices for group-based multi-trajectory modeling in boys (*n*=471).

**Supplementary Table S5.** Descriptive information on the severity of eating disorder psychopathology at baseline and last assessment in girls (*n*=427).

**Supplementary Table S6.** Descriptive information on the severity of eating disorder psychopathology at baseline and last assessment in boys (*n*=471).

**Supplementary Table S7.** Group labels and description for trajectory groups.

**Supplementary Method**

**Data analytic plan: Group-based multi-trajectory modeling (GBMTM)**

Group-based multi-trajectory modeling (Nagin et al., 2005) was employed to identify latent clusters of adolescents exhibiting similar trajectories in binge eating, dietary restraint, compensatory behaviors, and body mass index-standard deviation score (BMI-SDS). This method uses multinomial modeling with maximum likelihood estimation of model parameters. Prior to estimating multivariate models, separate univariate group-based trajectory models were fitted for each indicator to characterize their developmental course and to identify plausible polynomial orders for time (0–3; intercept only, linear, quadratic, cubic). A two-step procedure (Nagin et al., 2005, Nagin et al., 2024; Lu et al., 2022) was then implemented to determine the best-fitting trajectory model for up to seven classes. In the first step, the number of latent trajectories was determined by starting with a single trajectory and progressively increasing the number of trajectories, until the best-fitting model was identified. Model selection was based on four criteria (Klijn et al., 2017; Lu et al., 2022; Nagin et al., 2005): The Bayesian Information Criterion (BIC) closest to 0, the average posterior probability of assignment (APPA) >.70, the odds of correct classification (OCC) >5.0, and at least 5% of participants assigned to each group. Following the principle of parsimony, the model with the fewest trajectory groups that still captured distinctive features of the data was selected. In the second step, the shape of the trajectories for each indicator (binge eating, restraint, compensatory behaviors, BMI-SDS) was modeled using polynomials of order 0–3. For each candidate number of trajectory groups, polynomial orders were initially selected using a data-driven procedure in Stata that estimated alternative group-specific polynomial orders with the traj plugin (Jones & Nagin, 2013) and compared model fit according to the BIC. Subsequently, group-specific polynomial orders were refined primarily based on graphical inspection of the estimated trajectories and in light of the shapes obtained in prior univariate trajectory models. At each step, changes in BIC, relative group sizes, and overall classification quality were evaluated to ensure that the final models remained parsimonious and interpretable.

**Missing data**

The number of eligible, observed, and missing values by sex and age is reported in Supplemental Tables S1 and S2, with eligibility defined as participants attending the study center and meeting the analytic inclusion criteria. Age categories represent annual assessment windows (e.g., “10 years” = 9.50–10.49 years, “11 years” = 10.50–11.49 years, etc.). Among eligible participants, missingness for BMI-SDS was low across ages (range: 0.7–2.5%). In contrast, ED symptom data showed substantially lower availability at ages 10, 16, and 17 years, consistent with the Eating Disorder Examination-Questionnaire for Children (ChEDE-Q; Goldschmidt et al., 2007; Hilbert, 2016) being routinely administered only within the target age range (approximately 10.50–15.49 years). Thus, missing ED symptom data outside this age range primarily reflect non-routine administration rather than non-attendance. Given the low levels of nonresponse-missingness where measures were routinely collected, the missing-at-random assumption for maximum-likelihood estimation appears plausible.

**Supplementary Results**

**Model selection**

For girls, model selection favored a five-group solution with low-to-moderate polynomial orders (binge eating: 0, 1, 1, 1, 2; restraint: 0, 1, 1, 1, 2; compensatory behaviors: 0, 1, 1, 1, 1; BMI-SDS: 0, 1, 1, 1, 1). Classification quality was good, with mean and lowest APPA values ≥.92 and mean and lowest OCC values ≥19.55. The groups comprised 19.83%, 36.79%, 21.07%, 16.48%, and 5.84% of participants (all ≥5%). Two-, three-, and four-group models showed poorer fit (more negative BIC) and did not adequately capture the observed heterogeneity. Several alternative five-group specifications with different combinations of polynomial orders were evaluated but yielded more negative BIC values without improving APPA or OCC and were therefore not retained. Although a six- and seven-group solution achieved a less negative BIC, multiple specifications with varying polynomial orders consistently produced at least one class containing fewer than 5% of participants and did not meaningfully improve classification quality; six-group models were therefore rejected in favor of the more parsimonious five-group solution.

For boys, model selection favored a six-group solution with constant trajectories for all indicators (order=0). Classification quality was high considering mean and lowest APPA and OCC values ≥.88 and ≥18.89, respectively. The groups comprised 9.43%, 24.20%, 30.70%, 12.84%, 10.34%, and 12.48% of participants (all >5%). Alternative specifications that allowed higher-order (linear, quadratic, or cubic) terms for selected groups or indicators consistently resulted in more negative BIC values and did not improve classification quality, and were therefore not retained (see Table S2). Five-group models showed poorer fit (more negative BIC) and did not offer additional interpretive value. A seven-group model approached a less negative BIC but included a very small class (3.82%) and slightly reduced classification accuracy and was therefore rejected.

**Supplementary References**

Goldschmidt, A. B., Doyle, A. C., & Wilfley, D. E. (2007). Assessment of binge eating in overweight youth using a questionnaire version of the child Eating Disorder Examination with instructions. *International Journal of Eating Disorders, 40*, 460–467. https://doi.org/10.1002/eat.20387

Hilbert, A. (2016). Eating Disorder Examination-Questionnaire für Kinder. Deutschsprachige Übersetzung [Eating Disorder Examination-Questionnaire for children. German translation]. dgvt-Verlag.

Jones, B. L., & Nagin, D. S. (2013). A note on a Stata plugin for estimating group-based trajectory models. *Sociological Methods & Research, 42,* 608-613. https://doi.org/ 10.1177/0049124113503141

Klijn, S. L., Weijenberg, M. P., Lemmens, P., van den Brandt, P. A., & Lima Passos, V. (2017). Introducing the fit-criteria assessment plot–A visualisation tool to assist class enumeration in group-based trajectory modelling. *Statistical Methods in Medical Research, 26*, 2424-2436. https://doi.org/10.1177/0962280215598665

Lu, W. H., Guyonnet, S., Martinez, L. O., Lucas, A., Parini, A., Vellas, B., & de Souto Barreto, P. (2023). Association between aging-related biomarkers and longitudinal trajectories of intrinsic capacity in older adults*. GeroScience, 45*, 3409-3418. https://doi.org/ 10.1007/s11357-023-00906-2

Nagin, D. (2005). *Group-based Modeling of Development*. Harvard University Press. https://doi.org/10.4159/9780674041318.

Nagin, D. S., Jones, B. L., & Elmer, J. (2024). Recent advances in group-based trajectory modeling for clinical research. *Annual Review of Clinical Psychology, 20*, 285-305. https://doi.org/10.1146/annurev-clinpsy-081122-012416

**Table S1**

*Number of eligible, observed, and missing values for each indicator variable at each age in girls (n=427).*

|  | Eligible, *n* | ED symptoms | | |  | BMI-SDS | | |
| --- | --- | --- | --- | --- | --- | --- | --- | --- |
|  |  | Observed, *n* | Missing, *n* | Missing % |  | Observed, *n* | Missing, *n* | Missing % |
| 10 years | 201 | 32 | 169 | 84.1 |  | 196 | 5 | 2.5 |
| 11 years | 259 | 232 | 27 | 10.4 |  | 256 | 3 | 1.2 |
| 12 years | 299 | 271 | 28 | 9.4 |  | 296 | 3 | 1.0 |
| 13 years | 320 | 289 | 31 | 9.7 |  | 314 | 6 | 1.9 |
| 14 years | 280 | 273 | 7 | 2.5 |  | 276 | 4 | 1.4 |
| 15 years | 242 | 229 | 13 | 5.4 |  | 239 | 3 | 1.2 |
| 16 years | 195 | 66 | 129 | 66.2 |  | 192 | 3 | 1.5 |
| 17 years | 145 | 47 | 98 | 67.6 |  | 144 | 1 | 0.7 |

*Note.* *Eligible* denotes the number of participants who attended the study center at the respective age and met the analytic inclusion criteria (A2 cohort; one child per family; ≥2 assessment time points available within the observation window). *Observed* indicates available data for the respective measure; *Missing* = Eligible − Observed; Missing % = Missing / Eligible × 100. Age categories reflect annual assessment windows (e.g., “10 years” = 9.50–10.49 years; “11 years” = 10.50–11.49 years; etc.). ED symptoms were routinely administered only within the target age range 11–15 years, i.e., approximately 10.50–15.49 years; therefore, the high proportion of missing ED symptom data at ages 10, 16, and 17 years primarily reflect that the questionnaire was not routinely administered rather than non-attendance. BMI-SDS, Body mass index-standard deviation score; ED, Eating disorder.

**Table S2**

*Number of missing values for each indicator variable at each age in boys (n=471).*

|  | Eligible, *n* | ED symptoms | | |  | BMI-SDS | | |
| --- | --- | --- | --- | --- | --- | --- | --- | --- |
|  |  | Observed, *n* | Missing, *n* | Missing % |  | Observed, *n* | Missing, *n* | Missing % |
| 10 years | 231 | 36 | 195 | 84.4 |  | 229 | 2 | 0.9 |
| 11 years | 303 | 269 | 34 | 11.2 |  | 299 | 4 | 1.3 |
| 12 years | 350 | 307 | 43 | 12.3 |  | 343 | 7 | 2.0 |
| 13 years | 341 | 307 | 34 | 10.0 |  | 336 | 5 | 1.5 |
| 14 years | 320 | 303 | 17 | 5.3 |  | 315 | 5 | 1.6 |
| 15 years | 266 | 250 | 16 | 6.0 |  | 260 | 6 | 2.3 |
| 16 years | 210 | 59 | 151 | 71.9 |  | 208 | 2 | 1.0 |
| 17 years | 144 | 42 | 102 | 70.8 |  | 141 | 3 | 2.1 |

*Note.* *Eligible* denotes the number of participants who attended the study center at the respective age and met the analytic inclusion criteria (A2 cohort; one child per family; ≥2 assessment time points available within the observation window). *Observed* indicates available data for the respective measure; *Missing* = Eligible − Observed; Missing % = Missing / Eligible × 100. Age categories reflect annual assessment windows (e.g., “10 years” = 9.50–10.49 years; “11 years” = 10.50–11.49 years; etc.). ED symptoms were routinely administered only within the target age range 11–15 years, i.e., approximately 10.50–15.49 years; therefore, the high proportion of missing ED symptom data at ages 10, 16, and 17 years primarily reflect that the questionnaire was not routinely administered rather than non-attendance. BMI-SDS, Body mass index-standard deviation score; ED, Eating disorder.

**Table S3**

*Fit indices for group-based multi-trajectory modeling in girls (n=427).*

| No. of trajectory groups | Polynomial function order | BIC | APPA | | OCC | | Proportions in each trajectory group (%) | | | | | | |
| --- | --- | --- | --- | --- | --- | --- | --- | --- | --- | --- | --- | --- | --- |
|  |  |  | Mean | The lowest value across group(s) | Mean | The lowest value across group(s) | 1 | 2 | 3 | 4 | 5 | 6 | 7 |
| 1 | Binge eating (0)  Restraint (1)  Compensatory behaviors (1)  BMI-SDS (1) | -8122.83 | 1 | 1 | NA | NA | 100 | - | - | - | - | - | - |
| 2 | Binge eating (0, 0)  Restraint (1, 1)  Compensatory behaviors (1, 1)  BMI-SDS (1, 1) | -7383.65 | 0.97 | 0.96 | 32.73 | 27.56 | 51.52 | 48.48 | - | - | - | - | - |
| 3 | Binge eating (0, 0, 0)  Restraint (1, 1, 1)  Compensatory behaviors (1, 1, 1)  BMI-SDS (1, 1, 1) | -7110.12 | 0.95 | 0.95 | 55.44 | 20.00 | 31.62 | 50.12 | 18.27 | - | - | - | - |
| 4 | Binge eating (0, 0, 0, 0)  Restraint (1, 1, 1, 1)  Compensatory behaviors (1, 1, 1, 1)  BMI-SDS (1, 1, 1, 1) | -6963.82 | 0.94 | 0.91 | 83.52 | 15.07 | 29.27 | 39.58 | 15.93 | 15.22 | - | - | - |
| 5 | Binge eating (0, 0, 0, 0, 0)  Restraint (0, 0, 0, 0, 0)  Compensatory behaviors (1, 1, 1, 1, 1)  BMI-SDS (1, 1, 1, 1, 1) | -6873.87 | 0.94 | 0.91 | 214.18 | 21.15 | 19.67 | 35.36 | 19.20 | 19.91 | 5.85 | - | - |
| 5 | Binge eating (1, 1, 1, 2, 2)  Restraint (0, 1, 2, 2, 3)  Compensatory behaviors (0, 1, 2, 2, 3)  BMI-SDS (0, 1, 1, 1, 1) | -6866.48 | 0.93 | 0.92 | 62.45 | 19.30 | 19.96 | 36.88 | 21.00 | 16.35 | 5.81 | - | - |
| 5 | Binge eating (0, 1, 1, 2, 2)  Restraint (0, 1, 1, 2, 2)  Compensatory behaviors (0, 1, 1, 2, 2)  BMI-SDS (0, 1, 1, 1, 1) | -6853.82 | 0.93 | 0.92 | 61.43 | 19.54 | 19.81 | 36.74 | 21.15 | 16.46 | 5.84 | - | - |
| 5 | Binge eating (1, 1, 2, 2, 2)  Restraint (0, 1, 2, 2, 3)  Compensatory behaviors (0, 1, 2, 2, 2)  BMI-SDS (1, 1, 1, 1, 1) | -6849.14 | 0.93 | 0.90 | 76.93 | 29.72 | 17.83 | 32.60 | 22.02 | 16.27 | 11.27 | - | - |
| 5 | Binge eating (0, 0, 1, 1, 1)  Restraint (0, 0, 1, 1, 1)  Compensatory behaviors (0, 0, 1, 1, 1)  BMI-SDS (0, 0, 1, 1, 1) | -6847.58 | 0.93 | 0.90 | 82.38 | 31.53 | 15.40 | 31.05 | 23.98 | 16.86 | 12.72 | - | - |
| **5** | **Binge eating (0, 1, 1, 1, 2)**  **Restraint (0, 1, 1, 1, 2)**  **Compensatory behaviors (0, 1, 1, 1, 1)**  **BMI-SDS (0, 1, 1, 1, 1)** | **-6842.58** | **0.93** | **0.92** | **67.92** | **19.55** | **19.83** | **36.79** | **21.07** | **16.48** | **5.84** | - | - |
| 5 | Binge eating (0, 2, 2, 2, 2)  Restraint (0, 2, 2, 2, 3)  Compensatory behaviors (0, 3, 2, 2, 2)  BMI-SDS (0, 2, 2, 2, 2) | -6881.29 | 0.93 | 0.92 | 64.83 | 22.30 | 19.51 | 35.62 | 17.71 | 21.22 | 5.94 | - | - |
| 5 | Binge eating (0, 1, 1, 1, 1)  Restraint (0, 1, 2, 1, 3)  Compensatory behaviors (0, 3, 1, 1, 1)  BMI-SDS (0, 1, 2, 1, 1) | -6851.32 | 0.93 | 0.92 | 63.92 | 20.06 | 19.79 | 36.62 | 21.09 | 16.60 | 5.90 | - | - |
| 6 | Binge eating (0, 0, 0, 0, 0, 0)  Restraint (0, 0, 0, 0, 0, 0)  Compensatory behaviors (3, 3, 3, 3, 3, 3)  BMI-SDS (1, 1, 1, 1, 1, 1) | -6861.95 | 0.94 | 0.92 | 150.86 | 26.15 | 16.86 | 30.91 | 19.67 | 11.01 | 17.80 | 3.74 | - |
| 6 | Binge eating (1, 2, 1, 2, 2, 2)  Restraint (0, 0, 2, 2, 3, 3)  Compensatory behaviors (0, 2, 2, 2, 3, 3)  BMI-SDS (1, 1, 1, 1, 1, 1) | -6800.68 | 0.93 | 0.90 | 97.08 | 29.41 | 14.58 | 29.83 | 23.49 | 15.71 | 11.86 | 4.53 | **-** |
| 6 | Binge eating (0, 0, 1, 1, 2, 2)  Restraint (0, 0, 1, 1, 2, 2)  Compensatory behaviors (0, 0, 1, 1, 2, 2)  BMI-SDS (0, 0, 1, 1, 1, 1) | -6777.40 | 0.92 | 0.90 | 75.43 | 24.29 | 14.52 | 28.81 | 24.36 | 12.65 | 14.99 | 4.68 | - |
| 6 | Binge eating (0, 0, 1, 1, 2, 2)  Restraint (0, 0, 2, 2, 3, 3)  Compensatory behaviors (0, 0, 2, 2, 3, 3)  BMI-SDS (1, 1, 1, 1, 1, 1) | -6770.66 | 0.93 | 0.90 | 91.66 | 34.42 | 16.88 | 11.20 | 30.36 | 21.35 | 4.41 | 15.81 | **-** |
| 6 | Binge eating (1, 1, 1, 2, 2, 2)  Restraint (0, 1, 1, 1, 2, 2)  Compensatory behaviors (0, 1, 1, 1, 2, 2)  BMI-SDS (0, 1, 1, 1, 1, 1) | -6769.33 | 0.93 | 0.91 | 87.48 | 30.66 | 15.80 | 30.60 | 22.18 | 11.56 | 15.36 | 4.49 | **-** |
| 7 | Binge eating (0, 0, 0, 0, 0, 0, 0)  Restraint (0, 0, 0, 0, 0, 0, 0)  Compensatory behaviors (1, 1, 1, 1, 1, 1, 1)  BMI-SDS (1, 1, 1, 1, 1, 1, 1) | -6755.00 | 0.92 | 0.86 | 119.77 | 29.74 | 14.75 | 29.04 | 21.78 | 10.54 | 10.54 | 8.90 | 4.45 |

*Note.* The model that best fit our data is shown in bold. Polynomial function order: 0=intercept, 1=linear, 2=quadratic, 3=cubic.

APPA, average posterior probability of assignment; BIC, Bayesian information criterion; BMI-SDS, body mass index-standard deviation score; NA, not applicable; OCC, odds of correct classification.

**Table S4**

*Fit indices for group-based multi-trajectory modeling in boys (n=471).*

| No. of trajectory groups | Polynomial function order | BIC | APPA | | OCC | | Proportions in each trajectory group (%) | | | | | | |
| --- | --- | --- | --- | --- | --- | --- | --- | --- | --- | --- | --- | --- | --- |
|  |  |  | Mean | The lowest value across group(s) | Mean | The lowest value across group(s) | 1 | 2 | 3 | 4 | 5 | 6 | 7 |
| 1 | Binge eating (0)  Restraint (0)  Compensatory behaviors (0)  BMI-SDS (0) | NA | NA | NA | NA | NA | 100 | - | - | - | - | - | - |
| 2 | Binge eating (0, 0)  Restraint (0, 0)  Compensatory behaviors (0, 0)  BMI-SDS (0, 0) | -6795.03 | 0.97 | 0.96 | 31.39 | 18.62 | 58.39 | 41.61 | - | - | - | - | - |
| 3 | Binge eating (0, 0, 0)  Restraint (0, 0, 0)  Compensatory behaviors (0, 0, 0)  BMI-SDS (0, 0, 0) | -6502.61 | 0.96 | 0.95 | 70.47 | 19.38 | 26.96 | 50.32 | 22.71 | - | - | - | - |
| 4 | Binge eating (0, 0, 0, 0)  Restraint (0, 0, 0, 0)  Compensatory behaviors (1, 1, 1, 1)  BMI-SDS (0, 0, 0, 0) | -6300.34 | 0.94 | 0.91 | 93.74 | 19.14 | 11.68 | 38.00 | 34.61 | 15.71 | - | - | - |
| 5 | Binge eating (0, 0, 0, 0, 0)  Restraint (0, 0, 0, 0, 0)  Compensatory behaviors (0, 0, 0, 0, 0)  BMI-SDS (0, 0, 0, 0, 0) | -6227.86 | 0.92 | 0.87 | 76.58 | 22.59 | 12.38 | 38.96 | 16.91 | 14.59 | 17.15 | - | - |
| 5 | Binge eating (0, 2, 3, 3, 3)  Restraint (0, 3, 3, 3, 3)  Compensatory behaviors (0, 3, 3, 3, 3)  BMI-SDS (0, 3, 3, 3, 3) | -8827.71 | 0.88 | 0.85 | 28.12 | 8.69 | 33.81 | 44.70 | 0.55 | 16.02 | 4.91 | - | - |
| 5 | Binge eating (0, 1, 1, 1, 1)  Restraint (0, 1, 1, 1, 1)  Compensatory behaviors (0, 1, 1, 1, 1)  BMI-SDS (0, 3, 2, 1, 1) | -8697.17 | 0.83 | 0.69 | 16.43 | 8.78 | 36.88 | 37.27 | 13.23 | 8.54 | 4.08 | - | - |
| 5 | Binge eating (0, 0, 0, 2, 1)  Restraint (0, 0, 0, 0, 0)  Compensatory behaviors (0, 0, 0, 2, 1)  BMI-SDS (0, 0, 0, 0, 0) | -6252.38 | 0.92 | 0.90 | 60.99 | 18.23 | 8.62 | 34.22 | 26.59 | 19.44 | 11.12 | - | - |
| **6** | **Binge eating (0, 0, 0, 0, 0, 0)**  **Restraint (0, 0, 0, 0, 0, 0)**  **Compensatory behaviors (0, 0, 0, 0, 0, 0)**  **BMI-SDS (0, 0, 0, 0, 0, 0)** | **-6152.71** | **0.91** | **0.88** | **77.62** | **18.89** | **9.43** | **24.20** | **30.70** | **12.84** | **10.34** | **12.48** | **-** |
| 6 | Binge eating (0, 0, 0, 0, 0, 0)  Restraint (0, 0, 0, 0, 0, 0)  Compensatory behaviors (0, 0, 0, 0, 0, 0)  BMI-SDS (0, 0, 1, 1, 1, 1) | -6156.21 | 0.90 | 0.88 | 65.83 | 17.32 | 24.30 | 30.05 | 10.24 | 12.31 | 9.52 | 13.59 | - |
| 6 | Binge eating (0, 0, 0, 0, 1, 1)  Restraint (0, 0, 0, 0, 1, 1)  Compensatory behaviors (0, 0, 0, 0, 1, 1)  BMI-SDS (0, 0, 0, 0, 1, 1) | -6210.03 | 0.92 | 0.88 | 85.95 | 22.17 | 12.85 | 40.55 | 18.17 | 11.70 | 15.05 | 1.68 | - |
| 6 | Binge eating (0, 0, 2, 2, 2, 2)  Restraint (0, 0, 1, 1, 1, 2)  Compensatory behaviors (0, 1, 2, 2, 2, 2)  BMI-SDS (1, 1, 1, 2, 2, 2) | -6221.43 | 0.91 | 0.89 | 75.56 | 20.77 | 34.93 | 15.96 | 11.87 | 7.50 | 12.83 | 16.91 | - |
| 6 | Binge eating (0, 0, 1, 1, 3, 3)  Restraint (0, 0, 1, 1, 3, 3)  Compensatory behaviors (0, 0, 1, 1, 3, 3)  BMI-SDS (0, 0, 1, 1, 3, 3) | -8905.71 | 0.73 | 0.65 | 22.21 | 3.90 | 25.00 | 29.72 | 16.52 | 11.25 | 11.53 | 5.98 | - |
| 7 | Binge eating (0, 0, 0, 0, 0, 0, 0)  Restraint (0, 0, 0, 0, 0, 0, 0)  Compensatory behaviors (0, 0, 0, 0, 0, 0, 0)  BMI-SDS (0, 0, 0, 0, 0, 0, 0) | -6104.03 | 0.90 | 0.85 | 108.31 | 20.18 | 3.82 | 16.14 | 29.72 | 15.29 | 9.77 | 12.53 | 12.74 |

*Note.* The model that best fit our data is shown in bold. Polynomial function order: 0=intercept, 1=linear, 2=quadratic, 3=cubic.

APPA, average posterior probability of assignment; BIC, Bayesian information criterion; BMI-SDS, body mass index-standard deviation score; NA, not applicable; OCC, odds of correct classification.

**Table S5**

*Descriptive information on the severity of eating disorder and general psychopathology at baseline and last assessment in girls (n=427).*

| Variable | (1) Underweight | | (2) Normal weight | | (3) Early binge eating | | (4) Bulimic | | (5) Binge eating | |
| --- | --- | --- | --- | --- | --- | --- | --- | --- | --- | --- |
|  | *M (SD)/n* (%) | *n* | *M (SD)/n* (%) | *n* | *M (SD)/n* (%) | *n* | *M (SD)/n* (%) | *n* | *M (SD)/n* (%) | *n* |
| *Baseline assessment* | | | |  |  |  |  |  |  |  |
| ChEDE-Q ≥90^th^ percentile | 0 | 83 | 0 | 160 | 4 (4.4%) | 90 | 6 (8.7%) | 69 | 5 (20.0%) | 25 |
| SDQ self-report ≥90^th^ percentile | 9 (11.3%) | 80 | 4 (2.6%) | 156 | 9 (10.3%) | 87 | 10 (15.6%) | 64 | 3 (13.6) | 22 |
| SDQ parent-report ≥90^th^ percentile | 7 (9.0%) | 78 | 11 (7.2%) | 153 | 12 (13.6%) | 88 | 6 (8.8%) | 68 | 9 (36.0%) | 25 |
| *Last assessment* | | | |  |  |  |  |  |  |  |
| ChEDE-Q ≥90^th^ percentile | 0 | 83 | 0 | 160 | 0 | 90 | 13 (18.8%) | 69 | 6 (24.0%) | 25 |
| SDQ self-report ≥90^th^ percentile | 5 (6.0%) | 83 | 13 (8.2%) | 159 | 3 (3.3%) | 90 | 14 (20.3%) | 69 | 8 (32.0%) | 25 |
| SDQ parent-report ≥90^th^ percentile | 7 (8.9%) | 79 | 7 (4.7%) | 149 | 9 (10.3%) | 87 | 16 (23.5%) | 68 | 9 (36.0%) | 25 |

*Note.* ChEDE-Q, Eating Disorder Examination-Questionnaire for Children (global score); SDQ, Strengths and Difficulties Questionnaire (total difficulties score).

**Table S6**

*Descriptive information on the severity of eating disorder psychopathology at baseline and last assessment in boys (n=471).*

| Variable | (1) Underweight | | (2) Lower weight | | (3) Normal weight | | (4) Higher weight | | (5) Binge eating | | (6) Bulimic | |
| --- | --- | --- | --- | --- | --- | --- | --- | --- | --- | --- | --- | --- |
|  | *M (SD)/n* (%) | *n* | *M (SD)/n* (%) | *n* | *M (SD)/n* (%) | *n* | *M (SD)/n* (%) | *n* | *M (SD)/n* (%) | *n* | *M (SD)/n* (%) | *n* |
| *Baseline assessment* | | | |  |  |  |  |  |  |  |  |  |
| ChEDE-Q ≥90^th^ percentile | 0 | 45 | 0 | 113 | 0 | 145 | 1 (1.64%) | 61 | 4 (8.33%) | 48 | 4 (6.78%) | 59 |
| SDQ self-report ≥90^th^ percentile | 3 (6.8%) | 44 | 9 (8.6%) | 105 | 5 (3.6%) | 140 | 2 (3.4%) | 59 | 4 (8.3%) | 48 | 4 (6.9%) | 58 |
| SDQ parent-report ≥90^th^ percentile | 9 (20.5%) | 44 | 13 (11.7%) | 111 | 9 (6.5%) | 138 | 7 (12.1%) | 58 | 6 (13.3%) | 45 | 9 (15.5%) | 58 |
| *Last assessment* | | | |  |  |  |  |  |  |  |  |  |
| ChEDE-Q ≥90^th^ percentile | 0 | 45 | 0 | 113 | 0 | 145 | 0 | 61 | 0 | 48 | 2 (3.39%) | 59 |
| SDQ self-report ≥90^th^ percentile | 3 (6.7%) | 45 | 2 (1.8%) | 113 | 8 (5.6%) | 144 | 4 (6.6%) | 61 | 5 (10.6%) | 47 | 7 (11.9%) | 59 |
| SDQ parent-report ≥90^th^ percentile | 4 (9.5%) | 42 | 5 (4.6%) | 108 | 10 (7.1%) | 140 | 1 (1.8%) | 57 | 3 (7.7%) | 39 | 5 (9.8%) | 51 |

*Note.* ChEDE-Q, Eating Disorder Examination-Questionnaire for Children (global score); SDQ, Strengths and Difficulties Questionnaire (total difficulties score).

**Table S7**

*Group labels and description for trajectory groups*

| Trajectory group labels | | Description of trajectory groups |
| --- | --- | --- |
| Girls | Boys |  |
| *Underweight* | *Underweight* | Girls: Low ED symptoms and low age-adjusted BMI stable.  Boys: Low ED symptoms and low age-adjusted BMI stable. |
|  | *Lower weight* | Boys: ED symptoms absent and age-adjusted BMI at lower normal weight stable. |
| *Normal weight* | *Normal weight* | Girls: ED symptoms and age-adjusted BMI within normal weight range slightly increasing.  Boys: Low ED symptoms and age-adjusted BMI at normal weight stable. |
| *Early binge eating* | *Higher weight* | Girls: Early binge eating decreasing, low restraint and compensation stable, and age-adjusted BMI at higher normal weight increasing.  Boys: Low ED symptoms and age-adjusted BMI at higher normal weight stable. |
| *Binge eating* | *Binge Eating* | Girls: Binge eating, restraint, and compensation increasing to moderate levels, and age-adjusted BMI within the obesity range increasing.  Boys: Binge eating, restraint, and compensation stable at moderate levels, and age-adjusted BMI approaching obesity stable. |
| *Bulimic* | *Bulimic* | Girls: Binge eating, restraint, and compensation increasing to high levels, and age-adjusted BMI at normal weight stable.  Boys: Binge eating and restraint stable at moderate level, compensation stable at high level, and age-adjusted BMI at higher normal weight stable. |

*Note.* BMI, body mass index (kg/m²); ED, eating disorder.
